# The collaborative role of blockchain, artificial intelligence, and industrial internet of things in digitalization of small and medium-size enterprises

**DOI:** 10.1038/s41598-023-28707-9

**Published:** 2023-01-30

**Authors:** Abdullah Ayub Khan, Asif Ali Laghari, Peng Li, Mazhar Ali Dootio, Shahid Karim

**Affiliations:** 1 Department of Computer Science, Sindh Madressatul Islam University, Karachi, 74000 Pakistan; 2 Department of Computer Science and Information Technology, Benazir Bhutto Shaheed University Lyari, Karachi, 75660 Pakistan; 3 Software Collage, Shenyang Normal University, Shenyang, China; 4 School of Software Technology, Dalian University of Technology, Dalian, 116620 China; 5 Research & Development Institute of Northwestern Polytechnical University in Shenzhen, Shenzhen, 518057 China

**Keywords:** Computational science, Computer science

## Abstract

Due to digitalization, small and medium-sized enterprises (SMEs) have significantly enhanced their efficiency and productivity in the past few years. The process to automate SME transaction execution is getting highly multifaceted as the number of stakeholders of SMEs is connecting, accessing, exchanging, adding, and changing the transactional executions. The balanced lifecycle of SMEs requires partnership exchanges, financial management, manufacturing, and productivity stabilities, along with privacy and security. Interoperability platform issue is another critical challenging aspect while designing and managing a secure distributed Peer-to-Peer industrial development environment for SMEs. However, till now, it is hard to maintain operations of SMEs' integrity, transparency, reliability, provenance, availability, and trustworthiness between two different enterprises due to the current nature of centralized server-based infrastructure. This paper bridges these problems and proposes a novel and secure framework with a standardized process hierarchy/lifecycle for distributed SMEs using collaborative techniques of blockchain, the internet of things (IoT), and artificial intelligence (AI) with machine learning (ML). A blockchain with IoT-enabled permissionless network structure is designed called “B-SMEs” that provides solutions to cross-chain platforms. In this, B-SMEs address the lightweight stakeholder authentication problems as well. For that purpose, three different chain codes are deployed. It handles participating SMEs' registration, day-to-day information management and exchange between nodes, and analysis of partnership exchange-related transaction details before being preserved on the blockchain immutable storage. Whereas AI-enabled ML-based artificial neural networks are utilized, the aim is to handle and optimize day-to-day numbers of SME transactions; so that the proposed B-SMEs consume fewer resources in terms of computational power, network bandwidth, and preservation-related issues during the complete process of SMEs service deliverance. The simulation results present highlight the benefits of B-SMEs, increases the rate of ledger management and optimization while exchanging information between different chains, which is up to 17.3%, and reduces the consumption of the system’s computational resources down to 9.13%. Thus, only 14.11% and 7.9% of B-SME’s transactions use network bandwidth and storage capabilities compared to the current mechanism of SMEs, respectively.

## Introduction

The climate of global business and enterprises has changed the nature of development and related connectivity of SMEs. It is also because of the competitive fluctuates in the market and day-to-day challenges rising in the recent era, in which small and medium-sized businesses are performing only as receiving and adopting regulatory bodies. Manufacturing sectors contribute most of the ratio to economic development^1^. Whereas small and medium-sized enterprises, especially the production and manufacturing units, are on the developing agenda of various developed countries across the globe. While designing and creating units, one needs to consider the constraints of production and manufacturing development by size, scaling, and fund availability. In addition, owing to the amount, geographical reach, ability and capability of employees, and working intensity of entrepreneurs to drive their ideas to function. These are the main building blocks of SMEs that help in economic development^2^. According to the recent report on US economic cooperation and development (2021), all the connected stakeholders, including small companies countrywide and SMEs represent market fluctuations, which is more than 90% of overall enterprises^3,4^. However, SMEs generate half of the employment in the country and provide a turn ratio of more than 47% of the total increment of the business gross domestic product^5^. In a customer-driven environment, the production and manufacturing units are designed according to the procedures to respond back to mass customization using internet of things (IoT) technology, which provides a new pathway to value its customers.

Digitalization is a new paradigm that provides an opportunity to maintain SMEs in a better manner as compared to the traditional ones and transform them to allow more flexibility and agility. And so, SMEs' engines maintain the customer requirements by providing improved responsiveness accordingly. However, quality is a core component of customer-oriented infrastructure, being too upfront the transformation focus under the process of digitalization. The process hierarchy of creating SMEs is categorized as follows: (i) designing, (ii) planning, (iii) manufacturing, and (iv) performing functions or services.

While the quality of product, which is dispatched before to the customers of SMEs, is the fundamental objective to measure the favorable impact of production and manufacturing initially. Until there are no standard quality processes proposed, and no proper hierarchy currently being followed by the developers to design manufacturing units of SMEs that have evolved with the time and are converted to production. And so, these changes in the process hierarchy with developers maintain mass customization. The quality measurement has increasingly facilitated decisions based on the data collected in the dynamic time, from the marketplace and customer side. It is important to prioritize the processes that support SMEs' production and manufacturing and mitigate product development-related risks to design, plan, manufacture, develop, and perform functions within the defined resource constraints, such as less time and limited cost while fulfilling the customer requirement^6^. It is well noted, thus, to analyze the impact of digital manufacturing on quality measurement and to ensure that events of node transactions are functionally connected and synchronized at the same time.

**Figure 1:**
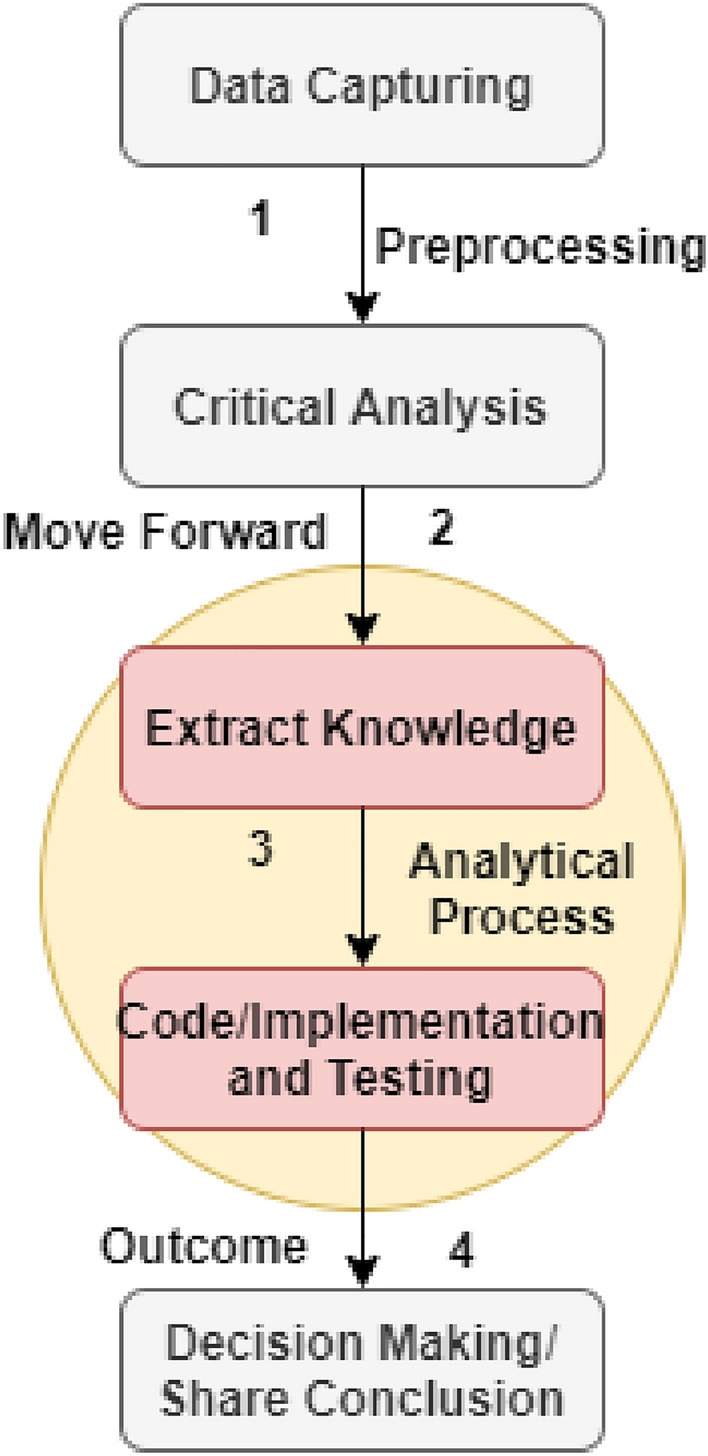
The current process of SMEs with IoT for data management and decision making.

Whereas the Internet of Things (IoT) and wireless sensor networks (WSN)-enabled SMEs' manufacturing process to change the traditional working operation of data gathering, examination, analysis, preservation, and present industrial and production records among the connected units^7^. It is possible to react directly and efficiently to customer-generated records with the use of the recent version of smaller batch sizes. Industrial IoT intends to corporate effectively with the processes of manufacturing SMEs, which is general to detect and recognize from the huge scale production. And so, because of industrial settings, where products are manufactured by indie with the help of a customer-specific approach. Mass customization is a concept that has drastically evolved in industrial production, need to design a cost-efficient method, which means the cost is equivalent to the mass products. A manufacturing unit based on the industrial IoT provides a modular architecture for replacing traditional systems^8^. It mainly focuses on the centralized server-based decision-making processes, and specific value-added evaluation is strictly limited in nature. However, SMEs and industrial IoT-enabled framework collaborates replace with versatile, reconfigure manufacturing and decentralized systems that provide effectiveness, responsiveness, strategic management, and building capability for decision making, as shown in Fig. 1 (using Draw.io for image generation).

In addition, the advancement in digital technology, such as IoT technology with SMEs provides improve productivity and income, captures market shares, creates brand awareness, mass customization, real-time feedback that enhances organizational implementation and necessary changes, helps in decision making, and evaluates customers sentiments^9^. On the other side, these highlighted factors pose a significant limitation in the SMEs-related transformation and digitalization. For this reason, various enterprises are unwilling and unsatisfied with speed cost, time, and related resource constraints because it consumes high engineering and investment initially, and the output is not cost-effective and satisfactory. However, to manage and optimize manufacturing and production-related data or transactions of SMEs in a ledger, various artificial intelligence (AI)-enabled machine learning (ML) techniques (supervised, unsupervised, and semi-supervised) are proposed^9^. Substantially, to expand the scope and context of AI development by tuning the mentioned SMEs' operations and integrated market fluctuations. Most importantly, the collaboration of AI with social computing performs a crucial role to develop a strong digital marketing strategies for SMEs by employing post and share mechanisms to spread awareness related to new manufacturing, production, and industrial development. Whereas performance and productivity depend to a certain degree. Therefore, it counts as long-term benefits.

In the current scenario, the centralized server-based network infrastructure is used for SMEs transactions acquisition towards deliverance. It affects directly SMEs' ledger integrity and privacy because of weak security, which means information is tracked and accessed easily, especially tracing customers' personal records through the Internet. Distributed ledger technology has been adopted widely in various small and medium-sized enterprise environments to avoid tampering and forgery^10–13^. Thus, blockchain is recently enabling SMEs to secure their running systems and process hierarchy to realize integrity, transparency, traceability, provenance, trustworthiness, and access ledger via distributed application (DApp). The existing SMEs are integrated with the collaborative approach of AI-blockchain, help to secure the process hierarchy, maintain customer-orientation strategy, encrypt exchanging information between customers and ecosystem, protected distributed node-to-node transactions, platform interoperability, and storage immutability. Furthermore, the individual event of node transaction is being stored before proper verification and validation using chain codes (smart contracts). A chain-like structure along with chronological order is designed that connects participating stakeholders in a public permissionless network to initiate SMEs’ transactions and exchange. This helps to achieve privacy and security for SMEs, which is hard to tamper with or forge and preserve optimized records in a secure storage container with hash-encrypted form.

The main research contributions and objectives of this paper are discussed as follows:

- This paper addresses the current process between customers and SMEs, such as manufacturing, production, and industrial units interconnective, including data gathering, management, and optimization. It is also analyzed that the implementation of digital technology with AI creates a new paradigm. Probably analysis of the impacts of SMEs on the countries’ economy highlights the benefits of adoption.
- In this paper, we highlight comparative research results related to digitalization, SMEs, industrial IoT, AI, blockchain, and their transformation conducted in Asia over the past few years.
- The outcome of the research in which a standard process hierarchy is derived, that led to managing customer relationships with SMEs at a secure, protected, and standard level. The proposed B-SMEs (a blockchain and AI-enabled distributed framework) provide a platform where the DApp is designed, created, and deployed for the sake of a transparent transactional environment. The main objectives are to handle SMEs-related automation, especially transaction verification and validation, exhaling, and sharing of resources among participants.
- With the development of blockchain distributed public permissionless networks integrated with AI provide a lightweight authentication mechanism that reduces the cost of computational resources along with network bandwidth and storage.
- There are three different chaincodes created and deployed for stakeholders’ registration, SME transactions and exchange updates, and information management and optimization of immutable storage. However, individual transactions are protected with the use of the NuCypher threshold re-encrypted throughout the deliverance. And so, the proof-of-work (PoW) and proof-of-stack (PoS) pre-defined ethereum consensus is adopted along with a digital signature to schedule, initiate, manage, and approve transactions and related information exchange among participating stakeholders.
- At last, this paper highlights a few of the open challenges, limitations, and issues involved in the implementation and deployment of DApp BSMEs in real-time, which will consider as future developments for technological maturity.

The remainder of this research paper is structured as follows: In Section “Related work”, there is various IoT, blockchain, and AI-enabled techniques involved in an industrial environment, and SMEs for better production and manufacturing are discussed. The primary knowledge of blockchain, IoT, and AI-enabling technologies is presented, along with the problem formulations and problem descriptions in Section “Fundamental knowledge and preliminaries”. B-SME, blockchain, IoT, and AI-enabled distributed framework, is proposed for smart SMEs development and related processes in Section “Proposed framework”. However, Section “Future direction” discusses the open implementation issues, challenges, and limitations and highlights future research directions with possible solutions. Finally, we conclude this paper in Section “Conclusion”.

## Related work

In digitalization, the concept of digital transformation is considered an efficient and effective business approach used to build improved enterprises' business practices compared to the traditional ones^14^. It reduces the impact of external limitations and introduces a great change in SMEs' operations by providing business strategies. While the adaptation of digital technology (DT), various challenging issues rises in both the sustainable economic development and social values of enterprises. However, the proper utilization of DT improves the regional economical-social conditions. To empower enterprises at every level, need to focus on the different phases of manufacturing growth, production, and sustainability. Due to this, there are various studies presented that investigated the types of issues, challenges, and limitations, and proposed different methods, such as moderating the role of entrepreneurial orientation, dynamic capability IoT strategy, resource-based views, etc. In this manner, we separated close methods, models, mechanisms, strategies, approaches, and conceptual architectures that use AI, industrial IoT, and blockchain-based modular framework to enhance the capabilities of SMEs. A few related works that highlight the research gaps involving the current systems of SMEs, and further discussion related to open changes popped up as follows, as mentioned in Table 1.

**Table 1:** Blockchain, AI, IIoT-enabled SMEs related literatures.

Research papers	Research development	Research benefits and outcomes	Research challenges, limitations, and issues
A role of moderating an entrepreneurial orientation to design a sustainable SMEs^14^	This study proposed practical and theoretical implications related to managers and ledgers of SMEs. The offered mechanism helps to transform their existing companies into digital enterprises. For this purpose, the study presented various theoretical implications, processes, and strategies that help in digital transformation	Volatile high velocity of business environment Dynamic capability review Resource-based review Benefits for startup SMEs as well	Cross sectional limitations External validity issues Hardly handle the generation to a large population Defect of context insensitivity The result of the proposed model in up to 68%
Automobile assembly model for design and implementation of SMEs using federated AI and blockchain technology^15^	The paper presented a new design for SMEs called Trust Threshold Limit (TTL) that helps moderate the existing use of embedded tools, sensors, energy, and cost of functions in the production and manufacturing process	Smart contract is involved in the automation of operational controls, events of nodes executions, and legalization of production and manufacturing Blockchain-based automobile assembly model	Trust threshold limit Limitation in federated learning of AI Consume more computational power to evaluate transactions Customization and graphical UI
An empirical study of SMEs: Analysis of the relationship between digital transformation and performance^16^	The authors of this paper presented an empirical analysis of the current performance of SMEs undergoing digital transformation and demonstrated a state-of-the-art review description	Interview method used The process of sustainable development is proposed Analysis digital transformation on financial performance	Cross chaining platform limitation Automation and deliverance related challenges Centralized data management and organization issues
A collaborative approach of knowledge, diffusion, and blockchain technology in SMEs^17^	This research presented a novel process hierarchy by adopting knowledge management perception, drawing on the distributed ledger technology, and highlighting the involvement of diffusion in intelligent SMEs and related transactions	A questionnaire-based evaluation Perform a logistic regression to analysis the determinate of futuristic digital transformation Use blockchain permissionless public network	Cost of data privacy and security Scope of data automation Interoperability platform issues Expensive data preservation on distributed storage
Fintech for SMEs sustainable business model and transformation^18^	This research highlighted the role of fintech in sector development under the influence of the fourth industrial revolution. In addition, this paper presented a novel framework using the ReSOLVE model for both the theoretical and practice advancement in SMEs	Sustainable business model Improve circular economy practices A conceptual framework is proposed Use Fintech-enabled ReSOLVE model	Linking fintech application Privacy and security issues SMEs data management and optimization limitations
The green blockchain for SMEs^19^	This paper proposed a secure distributed monitoring framework for the execution of core operations of SMEs using a blockchain public permissionless network	Eco-friendly systems Involvement of IoT technology Circular economy development Perform computationally intensive ML task	Regulatory and compliance issues Risk controlling procedure for data organization Data integrity and transparency-related challenges while connecting different SMEs

The integration of newly emerging technologies is ongoing, for example, AI, ML, federated learning, blockchain, hyperledger, IoT in industry, and green technology for SME assets that enables a modern collaborative approach^20–28^. The distributed ledger technology started gaining popularity as an evaluation of cryptocurrency, which is an automated distributed transaction platform that handles a number of requests without any centralized or third-party involvement. In this manner, SMEs and IoT collectively utilized blockchain as a secure infrastructure or a ledger that records events of node transactions and logs the changes in SMEs' expert status. However, there are various other benefits of using collaboratively blockchain, IoT, and AI, for the sake of improving the efficiency and capability of SMEs, cost-efficient transactions deliverance, and a secure information exchange environment. Therefore, we systematically reviewed various related studies that highlight the role of technology and its involvement in building a secure and protected SME environment (as discussed in Table 2).

**Table 2:** Systematic review of the technologies.

Categories	Research years and references									Our proposed work
	2013^20^	2014^21^	2015^22^	2016^23^	2017^24^	2018^25^	2019^26^	2020^27^	2021^28^	2022
Artificial intelligence	✓	✓	✓	✓		✓			✓	✓
Machine learning	✓	✓	✓	✓		✓			✓	✓
Internet of things					✓		✓	✓	✓	✓
Industrial and manufacturing process hierarchy						✓	✓		✓	✓
Process development	✓	✓		✓			✓	✓		✓
Customer oriented strategy	✓		✓	✓	✓	✓			✓	✓
Define relationship between customer and SMEs		✓				✓	✓	✓		✓
Information management and organization	✓						✓	✓	✓	✓
Blockchain involvement						✓	✓	✓	✓	✓
Privacy and security		✓		✓		✓			✓	✓

The list of comparison is mentioned as follows:

- Using artificial intelligence technique.
- Machine learning.
- Internet of things.
- Industrial and manufacturing process hierarchy.
- Process development.
- Customer oriented strategy.
- Define relationship between customer and SMEs.
- Information management and organization.
- Blockchain involvement.
- Addresses privacy and security.

## Fundamental knowledge and preliminaries

In this section, we discuss some fundamentals and preliminaries of the problem and formulate the possible solutions, which are as follows.

### Notation, problem formulation, and description

This paper discusses three different and major problems that have become the hot domain of SMEs nowadays, such as SMEs' data generation and process hierarchy, data management, and resource consumption, and privacy-protection preservation. These problems are tackled individually with various efficient solutions previously but integrating all these solutions into one environment is one of the challenging issues. Although, there is no standard mechanism available that integrates these technologies into a single platform with effectiveness and a reliable manner.

**Figure 2:**
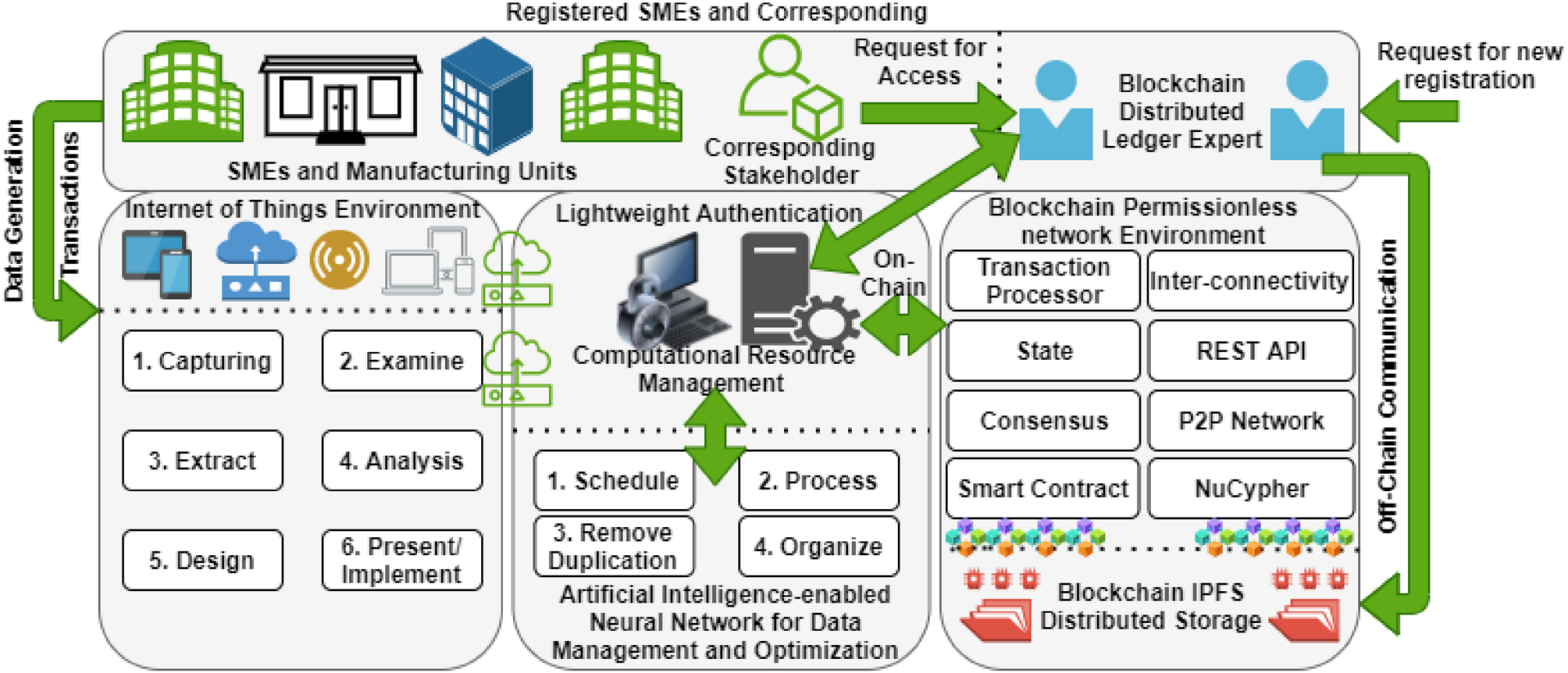
The proposed B-SMEs.

As we discussed, till now, there is no standard process hierarchy/lifecycle of SMEs proposed. In this regard, the proposed work identified this problem and addressed. And so, we proposed a B-SMEs standardized process hierarchy/lifecycle, as shown in Fig. 2. This lifecycle follows the standard procedure (according to the law of US economic cooperation and development) for IoT infrastructures to data gathering and management. Wireless sensor networks (WSN) associates with the IoT devices for the purpose to transmit data from one node to AI-enabled data resource management, as shown in Fig. 2. The process hierarchy of IoT-enabled devices for industrial, manufacturing, and production of SMEs are discussed as follows:

- Data capturing (capture-as-the-data-occur).
- Data examination.
- Data extraction.
- Data analysis.
- Design procedure to schedule process (priority bases).
- Implement a pathway for data traveling with the use of WSN.

For data management and optimization, we evaluate a classification mechanism that examines redundancy in data/transactions of SMEs and extracts the original ones while discarding duplication during the pre-verification process. This procedure helps computational resource management to reduce the computing cost and submit verified data in the ledger for further processes. For using this ML technique, especially artificial neural network (ANN) in the proposed B-SMEs to manage day-to-day transactions. In analyzing the ledger, ANN considers a breakthrough as it solves the data management, organization, and optimization. Before implementing, we identified a number of emerging loopholes involve in the distributed data management environment, such as data/transactions detection issues, and unstable recognition of files in the different nodes. Thus, to analyze these issues, we construct a data identification mechanism using machine learning and associate this with ANN, which efficiently extracts pattern, detect, recognize, and classify data files/transactions of SMEs. A self-correlation connects with the ANN to schedule logs for processing, along with that, it minimizes the risk of data capturing/loss by providing large-dimensional space for classification.

The notation of ANN with respect to the process of classification is discussed as follows:

- Weight (w’).
- Bias (b’).
- Input neurons (n’).
- Counter (c’).
- Single layer inputs (s’).
- Threshold (t’).
- Output neurons (o’).
- Activation function (σ).

For data verification process, weight is defined that objective is to create connection between the participating neurons in the ANN architectural design. Whereas single neuron holds a certain number of events od nodes data/transactions (values of data occurring). However, individual neuron of designed ANN assigns a unique values/label, then the input values are n_1_’, n_2_’, n_3_’, and n_s_’, with the assign weight w_1_’, w_2_’, w_3_’, and w_s_’, respectively.

The sum of inputs and weights, demonstrating the level of excitation, is as follows:

1 $$\mathrm{excitation fluctuation }=\sum_{c{^{\prime}}-1}^{s{^{\prime}}}w{^{\prime}}c{^{\prime}}*n{^{\prime}}c{^{\prime}}$$

If the range of the threshold fluctuates in an increasing manner of the proposed SMEs, then the output neuron (o’) = to the total (excitation fluctuation), which is defined in the expression discussed below:

2 $$\upsigma =\left\{\begin{array}{c}1 if \, total \, of \, {n}^{^{\prime}} \, and \, w{^{\prime}} is \, grater \, than \, is \, equal \, to ``t{^{\prime}}"\\ 0 if \, total \, of \, {n}^{^{\prime}} \, and \, {w}^{^{\prime}} \, is \, less \, than \, is \, equal \, to \, ``t{^{\prime}}"\end{array}\right.$$

However, the σ (sigma) is performed as the nonlinear function, where the t’ value = ‘0’, in which t’ down towards the minus side consider a negative sign.

Thus, that is the reason, we customize parameters of t’ by providing b’ and w’ values, such as w’0 = negative t’.

Therefore, a bias values b’0 = t’ is added to maintain ANN large-dimensional for data classification, such as if n’ value of n’ = 1 with formal inputs.

Then the output (o’) equation is expressed as follows:

3 $$\mathrm{o }'=\left\{\begin{array}{c}1 \, if \, total \, of \, n{^{\prime}} \, and \, w{^{\prime}} is \, grater \, than \, is \, equal \, to \, ``t{^{\prime}}"\\ 0 if \, total \, of \, n{^{\prime}} \, and \, w{^{\prime}} \, is \, less \, than \, is \, equal \, to \, ``t{^{\prime}}"\end{array}\right.$$

## Proposed framework

Figure 2 presents the working hierarchy of the proposed B-SMEs. An integrated blockchain and AI-enabled framework that is divided into three different folds. The first is the IoT process hierarchy, which is designed to collect, separate, examine, and analyze generated data or transactions of SMEs. After proper examination, we design a schedule to transmit data via wireless sensor networks and implement a managerial hierarchy that manages day-to-day transactions. Second, the AI compartment is split into two, such as computational resource management and AI-enabled neural network algorithm. A lightweight authentication is created in the middle, which aims to provide an automated capability to grant access to each applicational request after verification through the DApp. A new SME or corresponding registration is handled by the Blockchain Distributed Ledger Expert (BDLE). The BDLE is responsible for initiating new registration validation after a complete analysis of receiving requests and allows stakeholders to initiate transactions in the chain and exchange details. It is only a one-time process otherwise after registration it only needs to provide registration credentials to access the ledger. However, an Artificial Intelligence-enabled machine learning algorithm (such as an artificial neural network) is used that manages and optimizes data. This procedure discards duplication of data/transactions and organizes logs in a sequential order, which reduces the consumption of computational resources and preservation load at the same time.

Third, the blockchain permissionless public network (a peer-to-peer network with node interconnectivity) is deployed along with two different chain-of-communication, such as off-chain and on-chain. These two designed communication channels tackle a number of transactions that occur in the chain, for example, applicational requests, node-to-node activities, operational control, external communication, and information exchange. For instance, the on-chain receives internal transactional requests, which are implicitly handled. On the other side, off-chain communication tackles all explicit activities (outside of chain/cross-chain platform). However, the main objective of a blockchain transaction processor is to schedule a list of transactions, which is provided by outsourcing computation and execution, as shown in Fig. 2 (using Draw.io for image generation). For transaction protection and automation, we collaborate the concept of NuCypher threshold re-encryption with smart contracts and consensus policies, as discussed in Table 3. This is a new paradigm proposed in a public cryptographic encryption environment, which provides no cipher conversion. By the act of this, it reduces the load of computation by calculating hashes of individual transactions that occur in the B-SMEs. Whereas an InterPlanetary file storage system (IPFS) is used to store logs of individual transactions that occur in the B-SMEs chain. The purpose is to use this distributed immutable storage because provides ledger preservation facilities with minimal cost compared to other distributed storage, such as Filecoin. The major reason to use it is because it allows scalability and cost-efficient hierarchy (calculates usage of preservation) in a distributed manner.

**Table 3:**
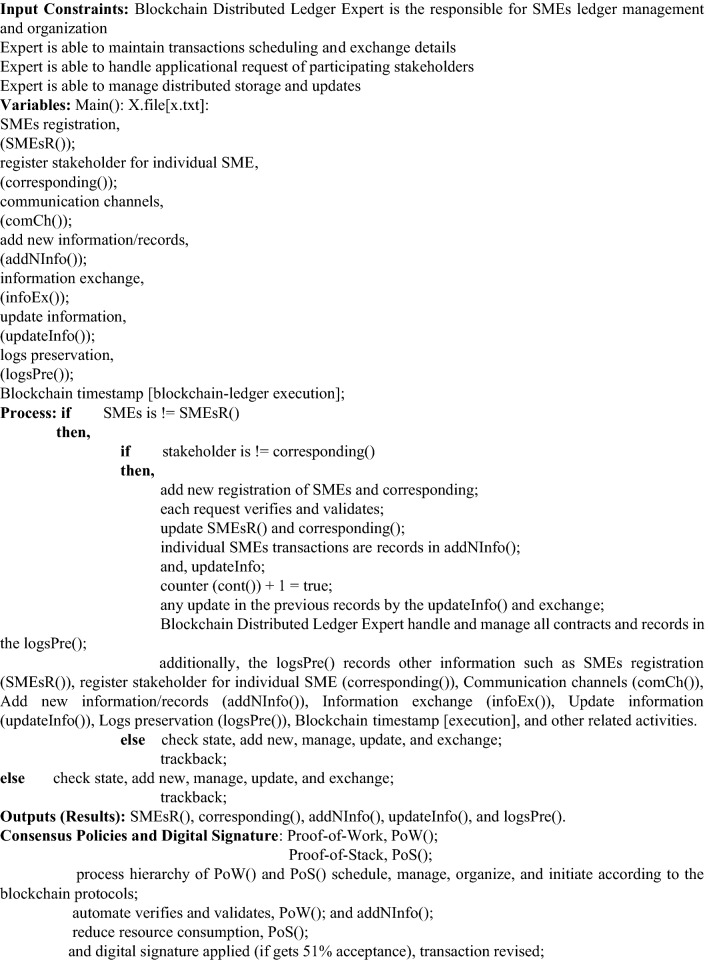
Chain codes implementation.

### Chain codes (smart contracts)

This paper presents three different chain codes and discusses their purposes to design, create, and deploy in this section. The list of contracts and consensus policies are as follows: (i) SMEsR(), (ii) corresponding(), (iii) addNInfo(), (iv) updateInfo(), (v) logsPre(), (vi) PoW(), and (vii) PoS().

The working operation of proposed B-SMEs is categorized into two different folds, such as chain codes and consensus policies with digital signature, as shown in Table 3. First, the aim of these designed contracts is to automate the verification and validation of new startups or SMEs registration along with the corresponding stakeholder. The SMEsR() and corresponding() contracts are initiated between the participating stakeholders and the system (B-SMEs DApp). These contracts are responsible to register new SMEs as per the policies created in B-SMES consensus (proof-of-work and proof-of-stack), as mentioned in Table 3. The function addNInfo() is created to record every transaction of all the participating SMEs and exchange. Whereas each transaction is recorded in the blockchain IPFS immutable storage and distributed on different nodes with protection using NuCypher threshold re-encryption. It also maintains B-SMEs ledger and optimizes while removing redundant logs with the help of ML-based neural network algorithm. However, the ledger updates after receiving update request from any of the corresponding SME for the purpose to revised transactions; it only possible when 51% votes received from the connected SMEs in accordance with the deployed protocol of digital signature of B-SMEs. After this, updateInfo() revised the logs and exchanged updated details among the participating stakeholders. While logsPre() is responsible for storing each and every activity that occur in the chain of B-SMEs.

### Results and discussion

**Figure 3:**
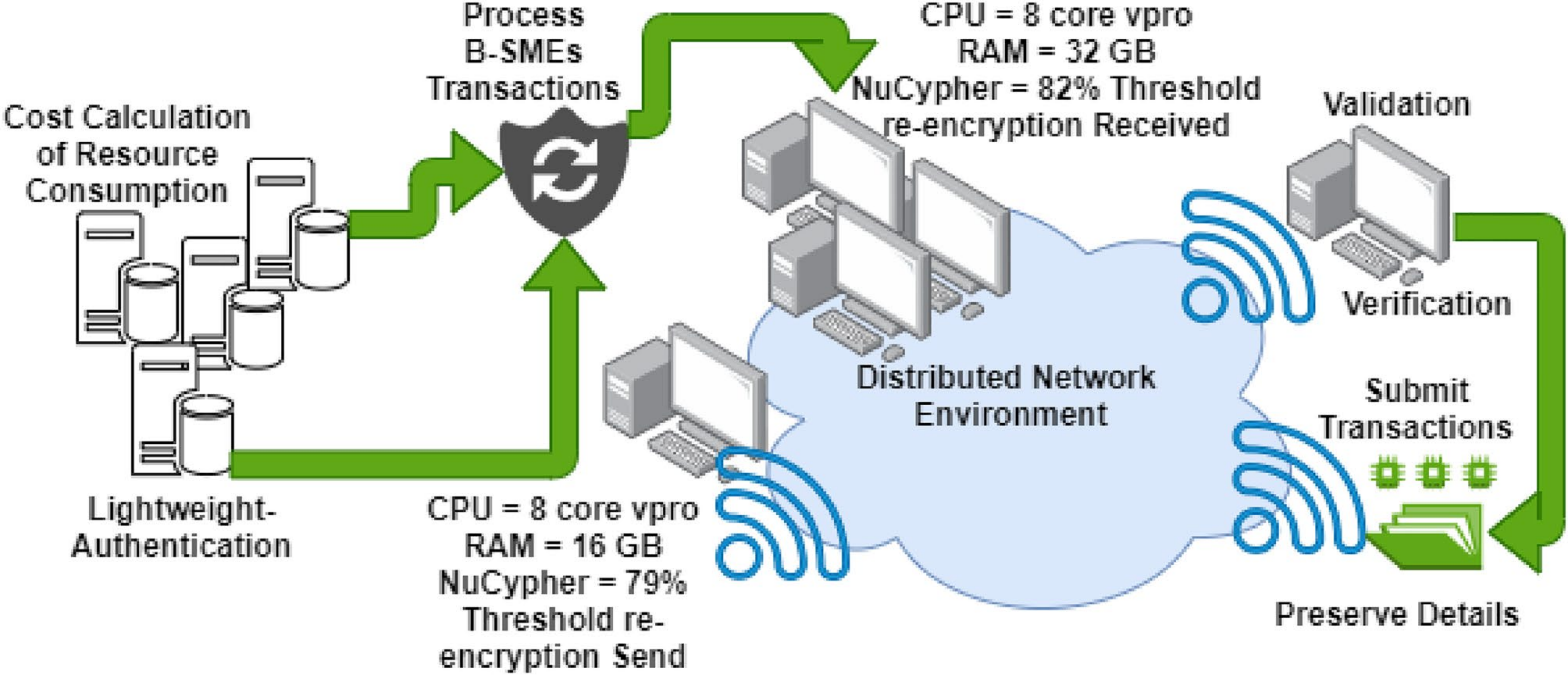
The simulation results of the proposed B-SMEs consensus for lightweight authentication, (**1**) shows the fluctuation of previous predefined consensus (PoW), and (**2**) shows the difference of adoptation of B-SMEs consensus (PoW and PoS).

After the problem description of data management and optimization using ANN (as discussed in Section “Notation, problem formulation, and description”). In this section, we present the working operation along with the simulation results of B-SMEs ledger data management, optimization, computational processing with lightweight authentication, and privacy preservation. For lightweight B-SMEs authentication, we connect a blockchain permissionless P2P public network with additional computing nodes to power the data processing, as shown in Fig. 3 (using Draw.io for image generation). Individual node is equipped with the eight-core i9 Evo processors (3.0 GHz with turbo boost). The systems run on Windows 11 with the blockchain docker and on their backend, the generic kernel is performed. These nodes are implemented with two different types of memories, such as static (limited in nature) and dynamic to preserve data/transactions during overall execution. However, the B-SMEs practically simulate on the simulated data with a few assumptions that are discussed as follows:

- A fixed size of node transactions = 4 MB.
- Local area network is designed with limited bandwidth of up to 1 Mb/s.
- Heterogenetic nodes interconnectivity is designed for intercommunication with two channels.

**Figure 4:**
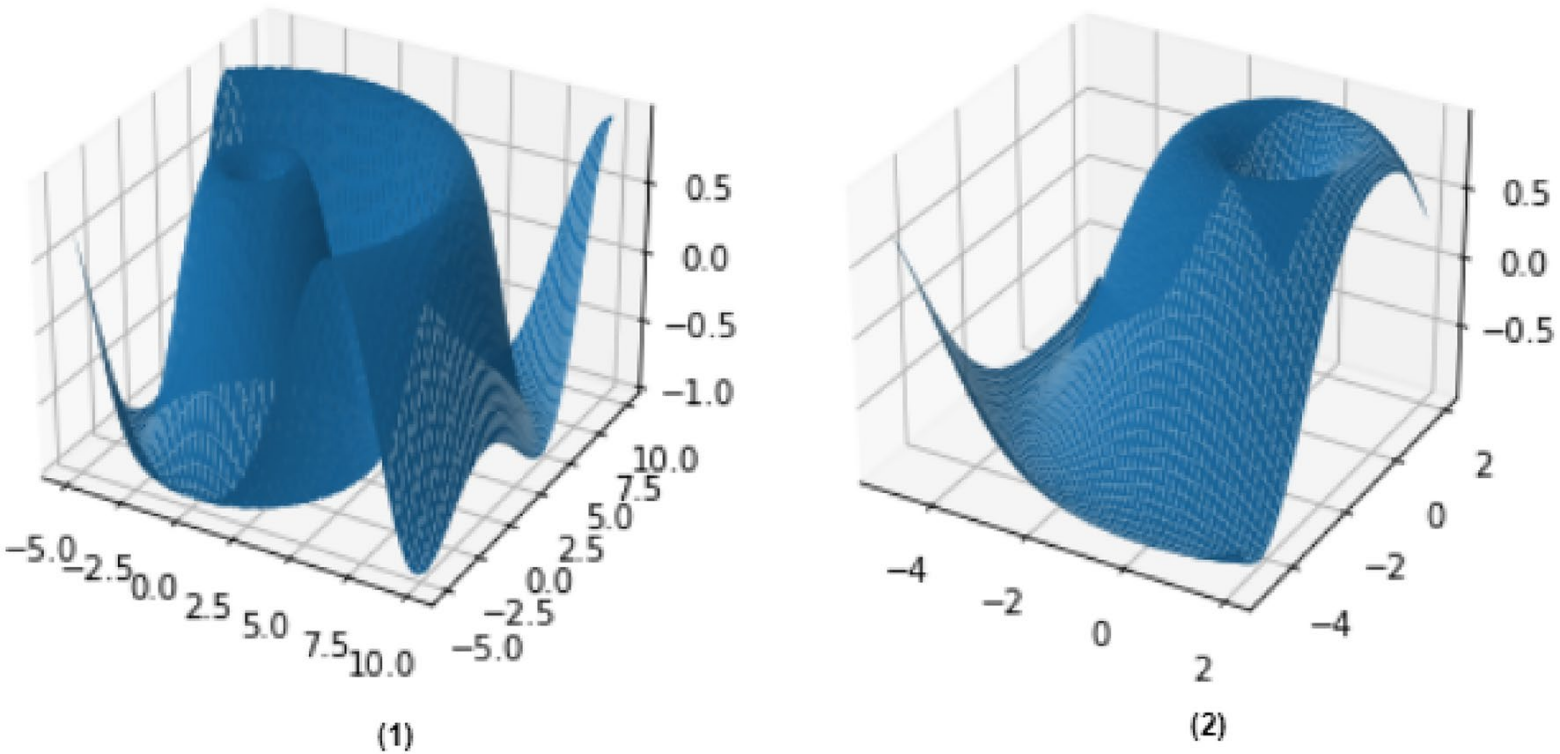
Lightweight authentication simulation assumptions.

For stakeholders’ lightweight authentication, we deployed smart contracts of BDLE to monitor the CPU usage and limit the systems’ computational energy, as shown in Figs. 2 and 3. The 3D simulation results (Fig. 4(1),(2)) of lightweight authentication show that current PoW and PoS consume more resources compared to the B-SMEs. The contrast between both the Figures illustrated the proposed B-SMEs customized consensus reduces the workload down to 9.13% and achieves remarkable cost reduction. However, with the adaptation of the B-SMEs consensus in real-time, we alleviate the usage of resource constraints in the complete process of user authentication and provide robust performance.

**Figure 5:**
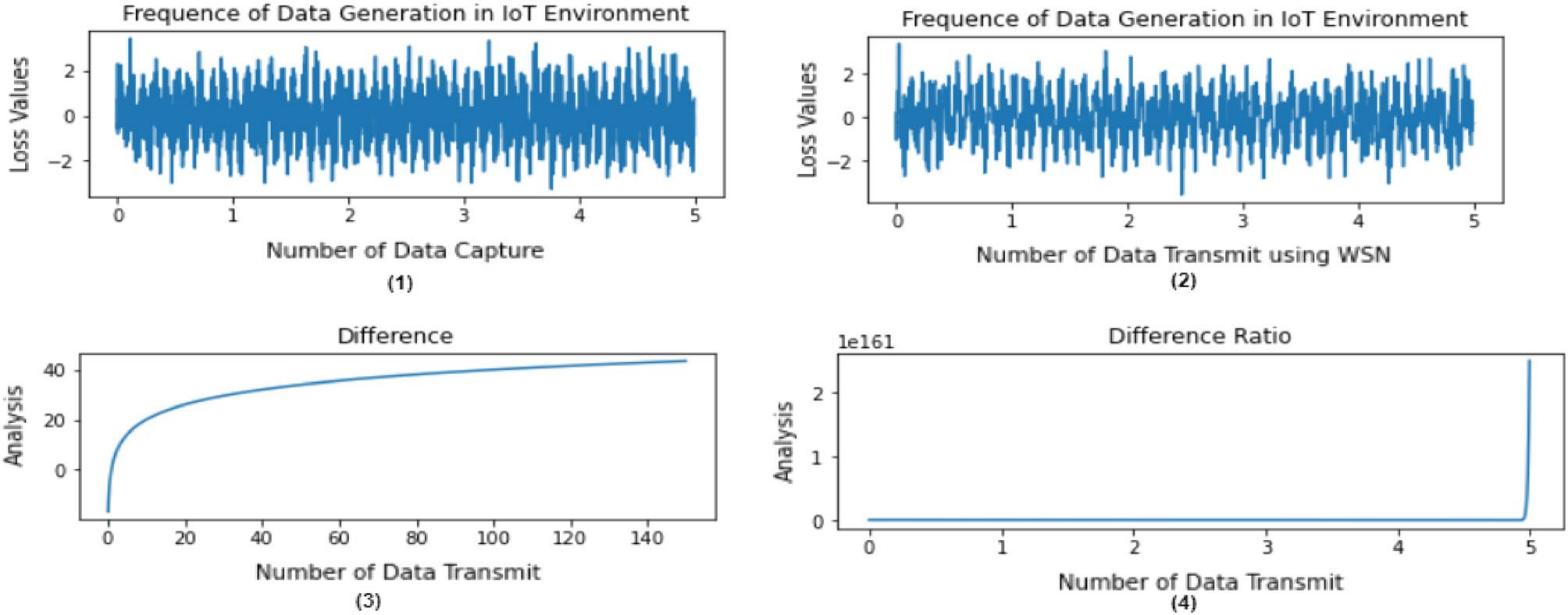
Frequency of data generation in IoT environment, (**1**) shows the number of data capturing, (**2**) shows the number of data transmitting, (**3**) shows the difference between the number of data transmission and analysis (**1**), and (**4**) shows the difference between the number of data transmission and analysis (**2**).

The analytical evaluation of IoT-enabled data/transactions collection and processing of SMEs is presented in Fig. 5. The result of this simulation is divided in to four folds; Fig. 5(1) and (2) illustrated the frequency of data generated by IoT devices, whose metrics is the number of data capture or transmitted using WSN and loss values (function). Whereas Fig. 5(3) and (4) presented the difference between the data transmitted and analysis, which is directly proportional to each other. This simulation helps in the ledger management and optimization while connected with the ANN. In fact, the integration of IoT with ANN reduces the delay and throughput and increase the response of data/transaction transmission. However, the proposed B-SME increases the rate of ledger management and optimization while exchanging information between different chains up to 17.3%.

**Figure 6:**
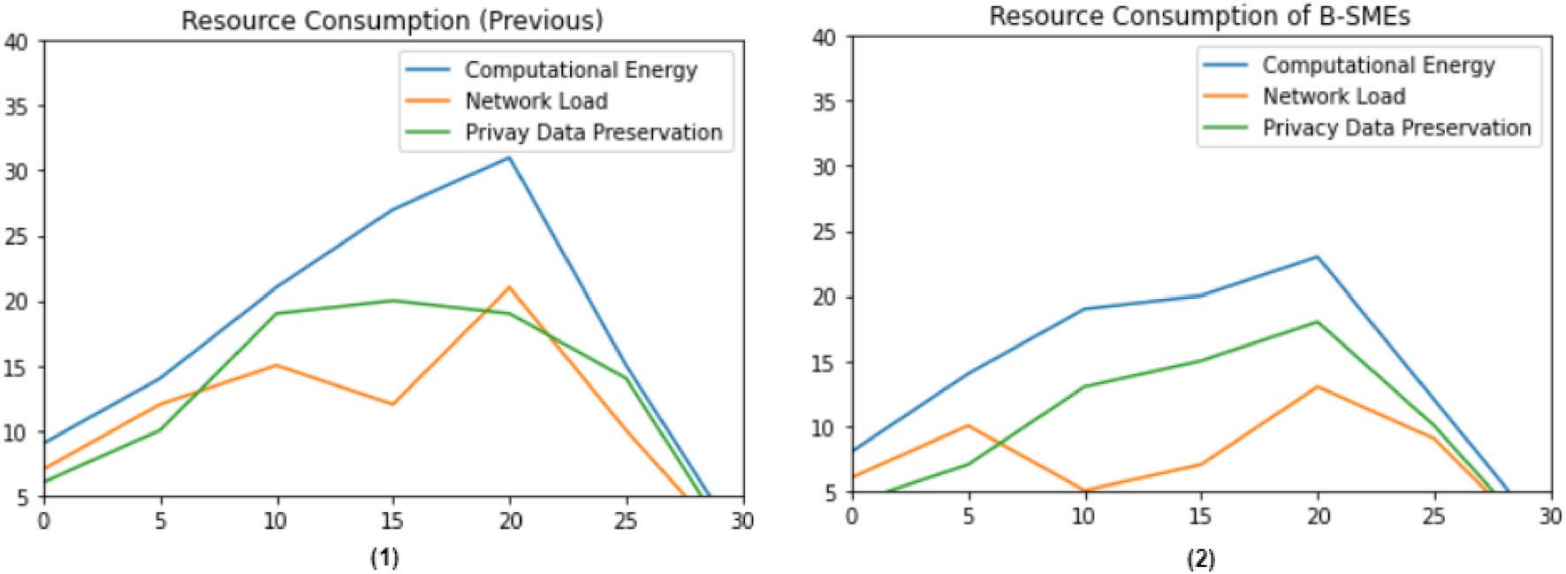
Cost of resource consumption (**1**) shows the graph of pervious work, and (**2**) shows the graph of the proposed B-SMEs.

Figure 6 shows the fluctuations occurs in the cost of resource consumption while transactions of SMEs are acquired to deliverance. The practical result of the proposed B-SMEs illustrated that the only 14.11% and 7.9% of B-SME’s transactions use network bandwidth and storage capabilities compared to the current mechanism of SMEs^30–33^, respectively.

In this context of comparison with other state-of-the-art, the paper presents a comparative table of a few related well-known studies that use AI, IoT, and blockchain-enabling technologies to improve the developments of small and medium-size enterprises' environment ^29^. However, the comparison results (in Table 4) show that the B-SMEs performed better and more efficiently. As the evaluation details are discussed in Table 4 as follows:

**Table 4:** Comparison with other state-of-the-art methods.

Other state-of-the-art	Contribution	Advantages and weakness	Our proposed system
Internationalization in India: A role of blockchain technology in small-medium enterprises^30^	This study proposed an advancement and integration model of business-to-consumer driven SMEs and robust them possess that perform more accurate operational deliverance. Substantially, it provides a holistic view compared to other states of the art	Survey on forty-three SMEs and collected records Regional specific Theoretical contribution Lack of data integrity, privacy, and security	In this attribute, we highlight the contribution, advancement, and benefits of the proposed B-SMEs. The list of improvement is discussed as follow: Integrated technologies: AI, blockchain, and IoT Chain: Chronological order (on-chain and off-chain) Infrastructure: Blockchain public network infrastructure Network: Distributed permissionless network Node Size: Fixed size 4-MB Encryption: NuCypher Re-encryption Consensus: Custom-build PoW and PoS Real-time usage: available to adoption for industrial use Reduce resource consumption: Down to 9.13% Efficiency: Increases up to 14.11% compared to others Accuracy: increases up to 7.9%
Small business emergence and adaptation for development of market growth^31^	This paper presented an identification process for selecting fintech and analytical algorithms. And so, designed SMEs which provide more reliability in terms of operations, control, strategic decision, and deliverance	Emerging markets and developing economies Big data involvement Data management, optimization, and preservation limitations	
Quality evaluation and potential impact in the development of SMEs^32^	The study focused on the development of SMEs authority, where all the startups and small businesses get the benefits of equality, such as industrial, manufacturing, production, and investment related	Small and medium development authority Compliance related issues in Pakistan Streamline automation of data handling issues	
A state-of-the-art method: the research on Industry 4.0 disruption in various industrial and manufacturing sections^33^	This research discussed the convergence of twelve disruptive technologies, such as AI, blockchain, IoT, cloud computing, big data, robotics, augmented reality, etc. And so this highlighted the need for extensive research in SMEs to increase the application of the disruptive	Level of discombobulation Cross chain platform limitations Retrofitting issues Increase cost of privacy and security	

## Future direction

In this context, the open research areas are presented in four different subsections; two are related to the digitalization and scope of SMEs in the future. And so, others are based on the customer relationship between SMEs-enabled privacy, security, and automation concerns using industrial IoT, AI, and blockchain.

### Blockchains and digitalization and scope of SMEs in the next decade

Nowadays, various financial institutions invest their assets in the development of blockchain infrastructure to create their all operations digitally and ensure security and robust efficiency. It is due to the increase acceleration in the development of information communication technology and digitalization that make SMEs and related activities more reliable^35,36^. For this infrastructure management, the designed system facilitates the process hierarchy of SMEs to manage reporting to top enterprises and regulatory authorities and provides convenience to combat money laundering. The distributed ledger technology confronts participating stakeholders at a chain-like structure, where individual transactions and information exchange can be seen (get through notification) and deliver every operation securely and smoothly along with logs preservation (immutable storage). However, the most suitable use of this collaborative technology with KYC compliance for robust industrial, manufacturing, and production of SMEs. Although, the usage of these integrated technologies will stimulate ideas about the current and the futuristic development of SMEs.

### Scalability of data privacy and security

It is highlighted that scalability of data management and the cost of privacy and security consider one of the critical challenges when designing and developing blockchain-enabled DApp^36^. In the domain of SMEs, the blockchain-based modular architecture collaborate with AI technology archives ledger integrity, system provenance, and transaction traceability by continuously stimulating distributed IPFS when every executing SMEs transactions and incorporating transmission details from the participating SMEs along with corresponding stakeholders. In this manner, the corresponding individual SME can check the details of financial growth, new participant, business logs, and related information with a click regardless of who direct the operations. There is no central authority or middleman available between the SMEs and regulatory body that manually verifies and validates a number of transactions. As developing this, the risk of tampering and forgery in the information exchange between participating stakeholders is reduced or almost restricted compared to the traditional ones. Thus, along with this, the blockchain permissionless public network avoids the concerns of participants while exchanging their operational logs with the regulatory and compliance authorities because of peer-to-peer connectivity.

### Distributed SMEs transaction and automation

In the B-SMEs environment, all the data manage and optimize in a distributed manner in the decentralized consortium, especially in public ledger using blockchain. With this development, the SMEs exchange critical information in a secure chain like structure via chronological order, for example, registration credentials, sale and purchase details, growth of financial assets, market fluctuations, startups values, and others^35,36^. The corresponding SMEs records core datils in blockchain distributed ledger and trace according to the requirement, such as futuristic employment and business growths, etc. These records are provided through the DApp with the use of blockchain public permissionless network-enabled environment, that is the reason it allows access to the participating SMEs to this sensitive and other-related details for robust economical contribution. However, the critical issues are to analysis and restrict repetitive data records, that consume more time to separate individual logs; for the act of this, it uses more power to computes. To overcome this situation, we design and create smart contracts (chain codes) along with consensus protocols to automate transaction verification and validation in accordance with the strategy of ledger records.

### Regulatory and cross platform issues

There are various challenges, limitations, and issues associated with the existing SMEs including the registration of startups, financial transactions exchange, and privacy, transparency in intercommunication, ledger maintenance in the centralized server-based preservation^34^. And so, it is relying on third-part data management and optimization tools with security solutions as well. In addition, to manage these issues, there are various tools, techniques, and applications used with the IoT technology to capture the records from portable devices^35,36^. Examine and analyze each log with different ML techniques to extract redundancy in the ledger and optimize it.

## Conclusion

This paper discusses the existing security and privacy procedure and separates the loopholes while SMEs are interconnected and exchange related information during the intercommunication between node-to-node over the centralized network. In this paper, we propose a B-SME, a collaborative technological framework using blockchain, IoT, and AI, that alleviates the resource usage of the collaborative technologies in the distributed network environment and create the system more reliable and efficient. This B-SMEs has implemented and deployed a secure process hierarchy of data management and optimization to be cost-effective and resource-efficient creating no problem in terms of the system’s provenance. It maintains efficient data integrity and a transparent, traceable, and reliable environment without affecting the container of blockchain docker. The experimental results shows that the exchanging information between interoperable chains increases up to 17.3%, where it reduces the consumption of the system’s computational resources down to 9.13%. Thus, only 14.11% and 7.9% of B-SME’s transactions use network bandwidth and storage capabilities compared to the current mechanism running for SMEs, respectively.

However, for this purpose, we created three different chain codes (smart contracts), along with customizing consensus policies (such as PoW and PoS) to automate and investigate individual transactions of SMEs with proper verification and validation process. In this manner, B-SMEs reduce the consumption of resources, such as the cost of computational energy, network load, and preservation. During each SMEs’ transactions acquisition to deliverance, the information of nodes is optimized and stored in blockchain ledger enabled IPFS according to the protocol designed in the chaincode, which is developed as per the requirement of a data structure for overall streamlining transmission, content management, and broadcasting. Substantially, all these SME-related transmissions (financial, social, economic, etc.) are scheduled and executed with two different distributed communication channels, such as on-chain and off-chain. Whereas on-chain communication handles all implicit operations. And so, off-chain manages operations explicitly. Finally, the experimental results illustrated that this proposed B-SMEs is a good candidate when implemented in the real-time industrial environment.

## Data Availability

The datasets generated and/or analyzed during the current study are not publicly available due to further investigation running on the same project for futuristic solutions but are available from the corresponding author on reasonable request.

## References

[1] Zutshi A, Mendy J, Sharma GD, Thomas A, Sarker T (2021). From challenges to creativity: Enhancing SMEs’ resilience in the context of COVID-19. Sustainability.

[2] Pereira V, Nandakumar MK, Sahasranamam S, Bamel U, Malik A, Temouri Y (2022). An exploratory study into emerging market SMEs’ involvement in the circular Economy: Evidence from India’s indigenous Ayurveda industry. J. Bus. Res..

[3] Adam NA, Alarifi G (2021). Innovation practices for survival of small and medium enterprises (SMEs) in the COVID-19 times: The role of external support. J. Innov. Entrep..

[4] Soni G, Kumar S, Mahto RV, Mangla SK, Mittal ML, Lim WM (2022). A decision-making framework for Industry 4.0 technology implementation: The case of FinTech and sustainable supply chain finance for SMEs. Technol. Forecast. Soc. Chang..

[5] Denicolai S, Zucchella A, Magnani G (2021). Internationalization, digitalization, and sustainability: Are SMEs ready? A survey on synergies and substituting effects among growth paths. Technol. Forecast. Soc. Chang..

[6] Pappas N, Caputo A, Pellegrini MM, Marzi G, Michopoulou E (2021). The complexity of decision-making processes and IoT adoption in accommodation SMEs. J. Bus. Res..

[7] Tudor AIM, Chițu IB, Dovleac L, Brătucu G (2021). IoT technologies as instruments for SMEs’ innovation and sustainable growth. Sustainability.

[8] Hansen EB, Bøgh S (2021). Artificial intelligence and internet of things in small and medium-sized enterprises: A survey. J. Manuf. Syst..

[9] Baabdullah AM, Alalwan AA, Slade EL, Raman R, Khatatneh KF (2021). SMEs and artificial intelligence (AI): Antecedents and consequences of AI-based B2B practices. Ind. Mark. Manage..

[10] Shaikh ZA, Ayub Khan A, Baitenova L, Zambinova G, Yegina N, Ivolgina N, Laghari AA, Barykin SE (2022). Blockchain hyperledger with non-linear machine learning: A novel and secure educational accreditation registration and distributed ledger preservation architecture. Appl. Sci..

[11] Khan AA, Laghari AA, Shafiq M, Cheikhrouhou O, Alhakami W, Hamam H, Ah-med Shaikh Z (2022). Healthcare ledger management: A blockchain and machine learning-enabled novel and secure architecture for medical industry. Hum. Centric Comput. Inf. Sci..

[12] Shaikh ZA, Khan AA, Teng L, Wagan AA, Laghari AA (2022). BIoMT modular infrastructure: The recent challenges, issues, and limitations in blockchain hyperledger-enabled e-healthcare application. Wirel. Commun. Mob. Comput..

[13] Khan AA, Laghari AA, Shaikh ZA, Dacko-Pikiewicz Z, Kot S (2022). Internet of Things (IoT) security with blockchain technology: A state-of-the-art review. IEEE Access.

[14] Vrontis D, Chaudhuri R, Chatterjee S (2022). Adoption of digital technologies by SMEs for sustainability and value creation: Moderating role of entrepreneurial orientation. Sustainability.

[15] Manimuthu A, Venkatesh VG, Shi Y, Sreedharan VR, Koh SCL (2022). Design and development of automobile assembly model using federated artificial intelligence with smart contract. Int. J. Prod. Res..

[16] Teng X, Wu Z, Yang F (2022). Research on the relationship between digital transformation and performance of SMEs. Sustainability.

[17] Bracci E, Tallaki M, Ievoli R, Diplotti S (2021). Knowledge, diffusion and interest in blockchain technology in SMEs. J. Knowl. Manage..

[18] Pizzi S, Corbo L, Caputo A (2021). Fintech and SMEs sustainable business models: Reflections and considerations for a circular economy. J. Clean. Prod..

[19] Hatzivasilis G, Ioannidis S, Fysarakis K, Spanoudakis G, Papadakis N (2021). The green blockchains of circular economy. Electronics.

[20] Schneider S, Spieth P (2013). Business model innovation: Towards an integrated future research agenda. Int. J. Innov. Manag..

[21] Asili GR, Hendi SS, Moallemi SA, Soofifard R, Kamali MR, Shavvalpour S, Vahabi MM (2014). Developing technical competency model to promote HRM in project-oriented organisations: A case for 3D petroleum system modelling in the Persian Gulf and Oman Sea (PEARL programme). Int. J. Prod. Qual. Manage..

[22] Roos C (2015). The Motivation and Factors Driving Crypto-Currency Adoption in SMEs.

[23] Ciuriak, D. *TPP's Business Asymmetries: Megaregulation and the Conditions of Competition Between MNCs and SMEs*. SSRN 3472317 (2016).

[24] Box S, Lopez-Gonzalez J (2017). The future of technology: Opportunities for ASEAN in the digital economy. Glob. Megatrends.

[25] Serrano-Calle, S., Robles, T., Martín, D. & Mateos, R. *Digitalization of Operations Management with Emotional and Intelligence Tools. Blockchain and IoT integration, the last disruption?* (2018).

[26] Li Z, Guo H, Wang WM, Guan Y, Barenji AV, Huang GQ, McFall KS, Chen X (2019). A blockchain and automl approach for open and automated customer service. IEEE Trans. Ind. Inf..

[27] Wong L-W, Leong L-Y, Hew J-J, Tan GW-H, Ooi K-B (2020). Time to seize the digital evolution: Adoption of blockchain in operations and supply chain management among Malaysian SMEs. Int. J. Inf. Manage..

[28] Chatterjee S, Chaudhuri R, Vrontis D, Basile G (2021). Digital transformation and entrepreneurship process in SMEs of India: A moderating role of adoption of AI-CRM capability and strategic planning. J. Strategy Manag..

[29] Pfister P, Lehmann C (2021). Returns on digitisation in SMEs: A systematic literature review. J. Small Bus. Entrep..

[30] Rakshit S, Islam N, Mondal S, Paul T (2022). Influence of blockchain technology in SME internationalization: Evidence from high-tech SMEs in India. Technovation.

[31] Akpan IJ, Udoh EAP, Adebisi B (2022). Small business awareness and adoption of state-of-the-art technologies in emerging and developing markets, and lessons from the COVID-19 pandemic. J. Small Bus. Entrep..

[32] Ahmad I, Serbaya SH, Rizwan A, Mehmood MS (2021). Spectroscopic analysis for harnessing the quality and potential of gemstones for small and medium-sized enterprises (SMEs). J. Spectrosc..

[33] Bongomin O, Yemane A, Kembabazi B, Malanda C, Mwape MC, Mpofu NS, Tigalana D (2020). Industry 4.0 disruption and its neologisms in major industrial sectors: A state of the art. J. Eng..

[34] Khan AA, Shaikh AA, Laghari AA (2022). IoT with multimedia investigation: A secure process of digital forensics chain-of-custody using blockchain hyperledger sawtooth. Arab. J. Sci. Eng..

[35] Khan AA, Laghari AA, Shafiq M, Awan SA, Gu Z (2022). Vehicle to everything (V2X) and edge computing: A secure lifecycle for UAV-assisted vehicle network and offloading with blockchain. Drones.

[36] Khan AA, Laghari AA, Gadekallu TR, Shaikh ZA, Javed AR, Rashid M, Estrela VV, Mikhaylov A (2022). A drone-based data management and optimization using metaheuristic algorithms and blockchain smart contracts in a secure fog environment. Comput. Electr. Eng..

